# Nitro and Other Electron Withdrawing Group Activated Ruthenium Catalysts for Olefin Metathesis Reactions

**DOI:** 10.1002/anie.202008150

**Published:** 2020-12-03

**Authors:** Anna Kajetanowicz, Karol Grela

**Affiliations:** ^1^ Laboratory of Organometallic Synthesis Faculty of Chemistry Biological and Chemical Research Centre University of Warsaw Żwirki i Wigury 101 02-089 Warsaw Poland; ^2^ Institute of Organic Chemistry Polish Academy of Sciences Kasprzaka 44/52 01-224 Warsaw Poland

**Keywords:** active pharmaceutic ingredients (APIs), olefin metathesis, process optimization, ruthenium catalysts, total synthesis

## Abstract

Advanced applications of the Nobel Prize winning olefin metathesis reaction require user‐friendly and highly universal catalysts. From many successful metathesis catalysts, which belong to the two distinct classes of Schrock and Grubbs‐type catalysts, the subclass of chelating‐benzylidene ruthenium complexes (so‐called Hoveyda–Grubbs catalysts) additionally activated by electron‐withdrawing groups (EWGs) provides a highly tunable platform. In the Review, the origin of the EWG‐activation concept and selected applications of the resulting catalysts in target‐oriented synthesis, medicinal chemistry, as well as in the preparation of fine‐chemicals and in materials chemistry is discussed. Based on the examples, some suggestions for end‐users regarding minimization of catalyst loading, selectivity control, and general optimization of the olefin metathesis reaction are provided.

## Graphical abstract

Chelating‐benzylidene ruthenium complexes (so‐called Hoveyda–Grubbs catalysts) activated by electron‐withdrawing groups (EWGs) are finding practical applications in target‐oriented synthesis, medicinal chemistry, the preparation of fine‐chemicals, and in materials chemistry.

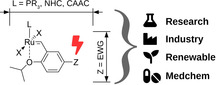

## 1 Olefin Metathesis Reaction and Established Catalysts Types

**Scheme 1:**
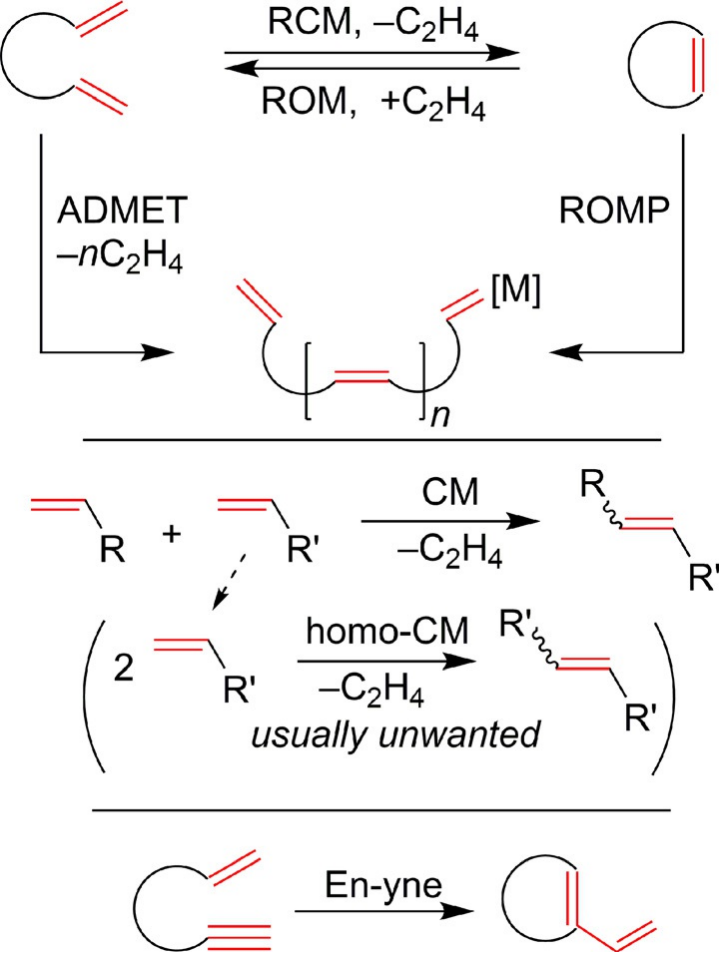
Basic types of olefin metathesis. RCM=ring‐closing metathesis; ROM=ring‐opening metathesis; ADMET=acyclic diene metathesis; ROMP=ring‐opening metathesis polymerization; CM=cross‐metathesis.

Olefin metathesis is a rising green technology that has changed the way chemists design and construct advanced organic architectures under mild conditions from simple starting materials (Scheme 1).[^1^, ^2^, ^3^] For the explanation of the mechanism of the olefin metathesis reaction and the landmark discovery of well‐defined transition‐metal‐carbene catalysts, Yves Chauvin, Robert H. Grubbs, and Richard R. Schrock were collectively awarded the 2005 Nobel Prize in Chemistry.[^4^, ^5^, ^6^, ^7^, ^8^, ^9^] This transformation has found a great deal of success in the fields of natural product synthesis, fine‐chemicals, medicinal chemistry, and materials science. Recently, the metathesis reaction has shown great promise in the context of utilizing renewable resources. Furthermore, it fits very well with the European Circular Economy and other sustainable production policies, as it can be used for the transformation of non‐edible oils in ethenolysis‐based bio‐refineries, waste recycling, in the production of polymeric composite materials for wind farms, environment‐friendly crop‐protection agents based on pheromones, etc.^[10]^

**Figure 1:**
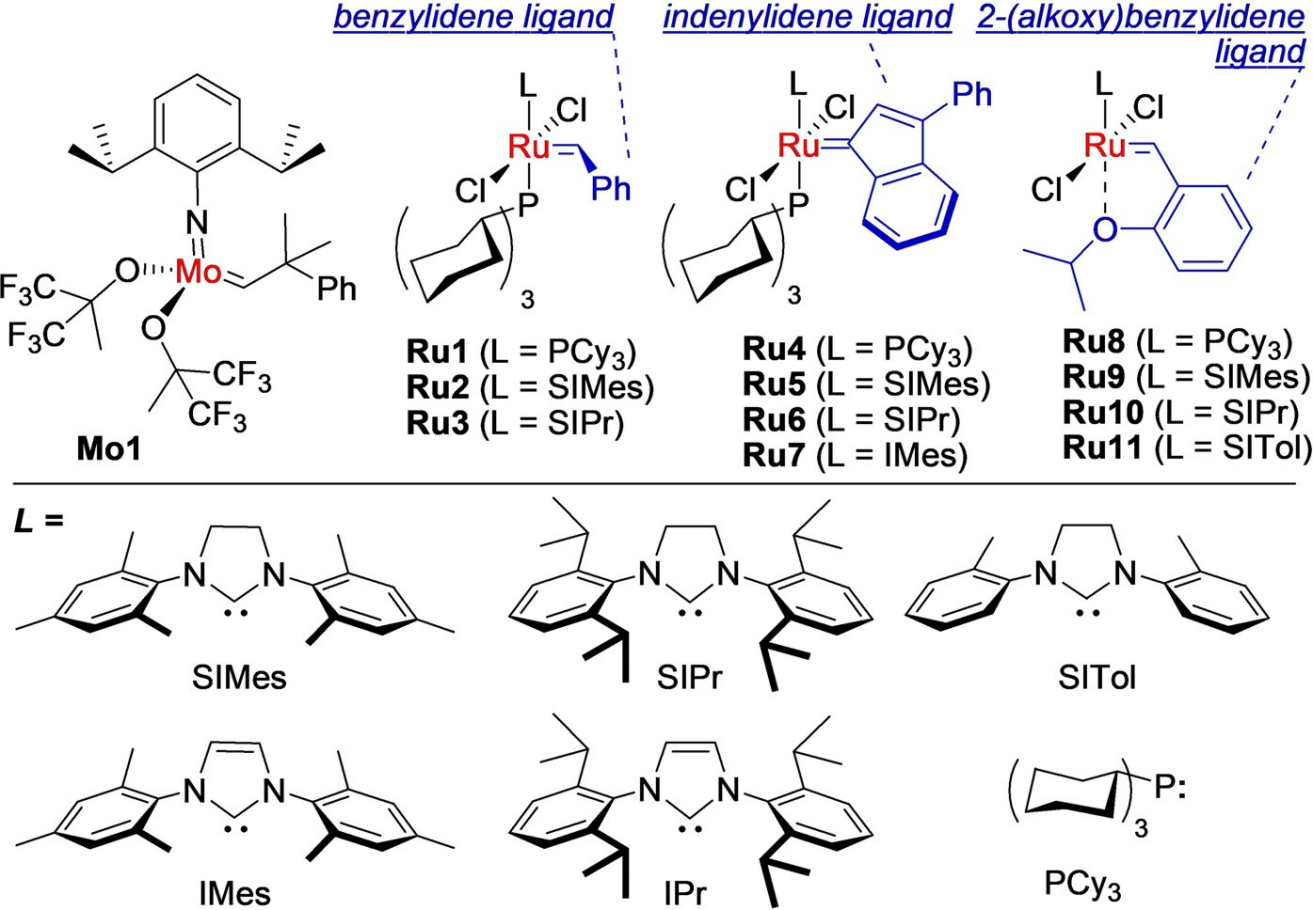
Selected Mo and Ru metathesis catalysts.

This enormous technological progress was possible thanks to modern well‐defined olefin metathesis catalysts.^[11]^ Among the transition‐metal complexes that catalyze olefin metathesis, ruthenium benzylidenes and indenylidenes (Figure 1) have proved to be highly practical, because of their air, moisture, and polar functional group tolerance, as well as their synthetic versatility. Olefin metathesis has been the subject of numerous general and specialized reviews,[^12^, ^13^, ^14^, ^15^, ^16^, ^17^] and the reader is asked to refer to the recently published books on metathesis, as they can provide a well‐organized view on this field.[^1^, ^2^, ^3^]

## 2 The EWG‐Activation Concept, Early Observations, and Consequences

**Figure 2:**
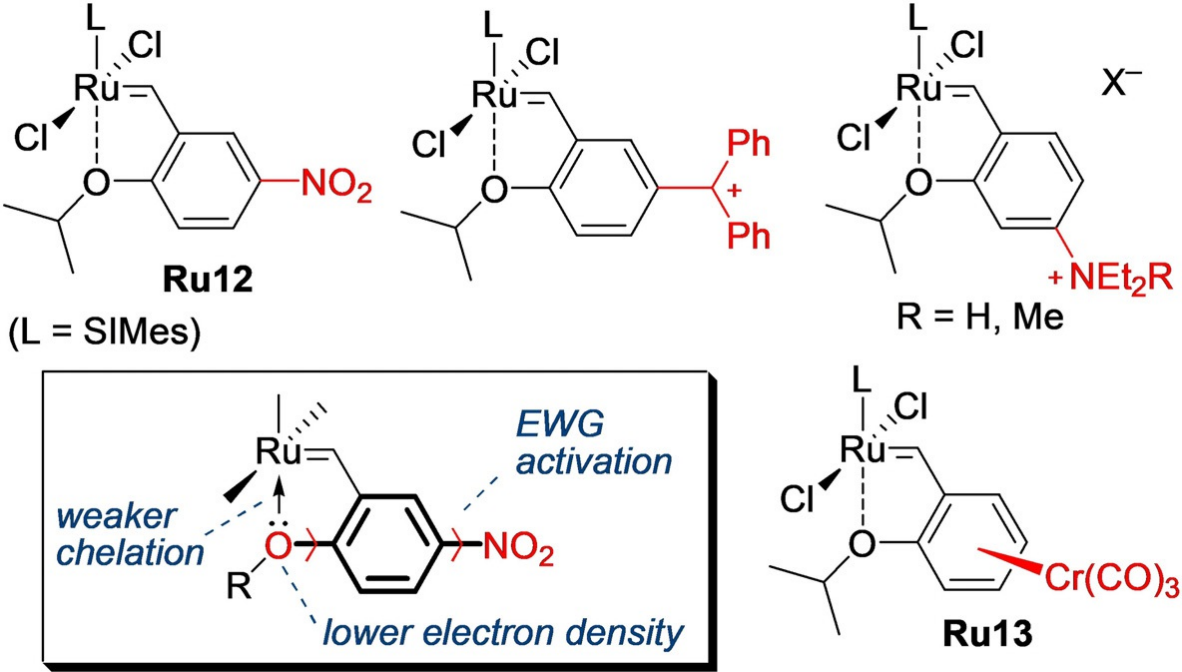
Selected EWG‐activated catalysts and proposed explanation of the effect. L=SIMes.

**Figure 3:**
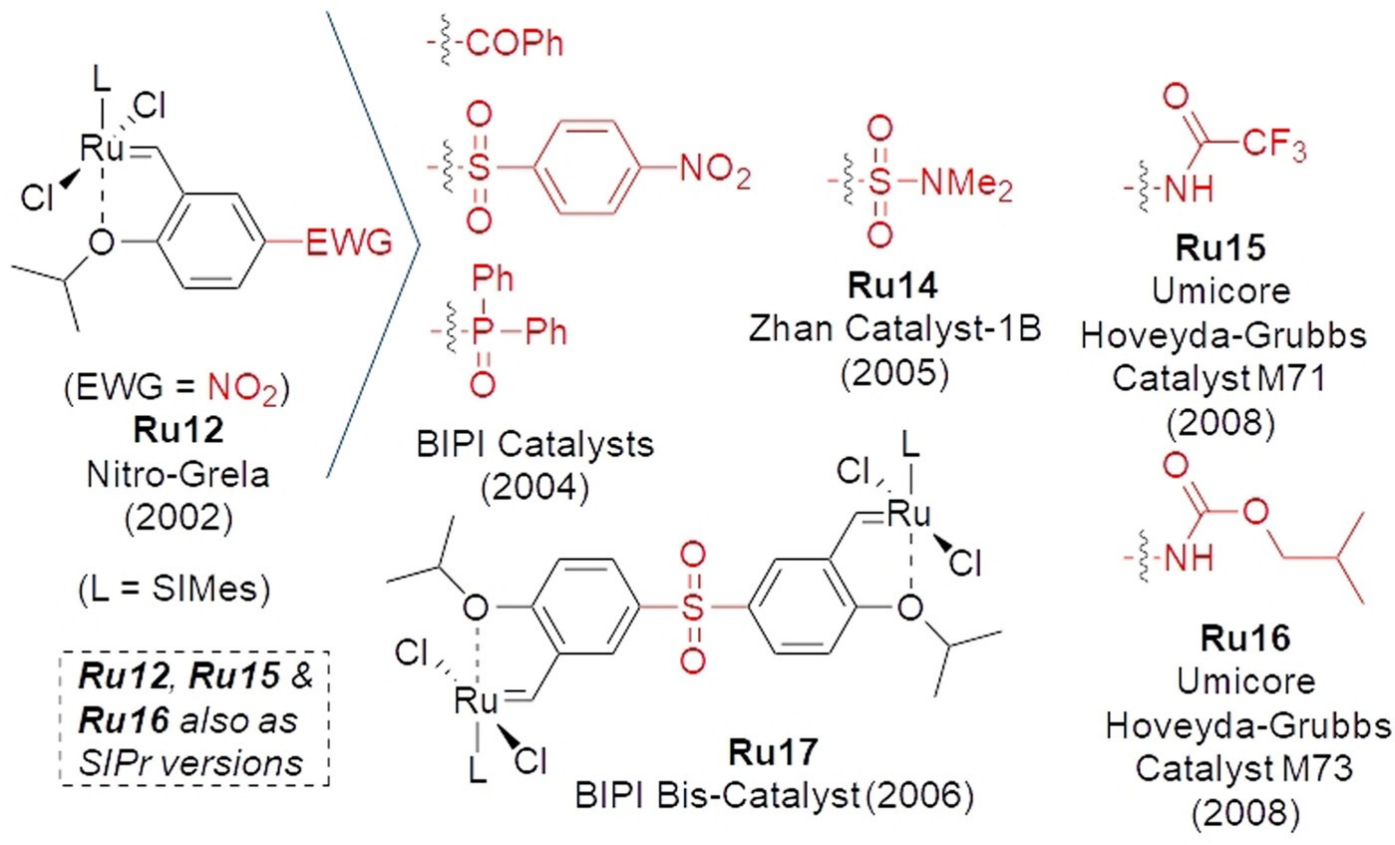
Selected commercial catalysts originating from the concept of EWG‐activation of **Ru12**.

Historically, the first Hoveyda–Grubbs catalysts^[18]^ that were substituted in the 2‐(alkoxy)benzylidene ligand with groups such as ‐OC(O)R and ‐Br were made in an attempt to immobilize the parent catalysts **Ru8** and **Ru9**.[^19^, ^20^] Although the idea of using these functional groups (being just anchors for immobilization) to control the initiation rate of the homogeneous catalysts was not considered at that time, the key observation was disclosed in 2002 when the strongly electron‐withdrawing nitro group was installed *para* to the chelating isopropoxy fragment of **Ru9**.[^21^, ^22^] Unexpectedly, this small structural alteration led to a large change in the activity: the nitro‐activated **Ru12** (Figure 2) was found to be visibly more active than the parent catalyst, initiated at 0 °C, and gave very good results in a number of challenging metathesis reactions.[^21^, ^22^] This observation was found to be true for many other electron‐withdrawing substituents, such as ‐SC_4_F_9_, ‐SO_2_Ar, ‐C(O)R and ‐P(O)R_2_,[^23^, ^24^, ^25^] as well as ‐SO_2_‐ (**Ru17**, Figure 3), placed in the *para* or *meta* position relative to the chelating oxygen atom.[^24^, ^26^] In fact, the same effect was observed with quaternary ammonium groups,^[27]^ or even protonated amines and the in situ generated carbocations^[28]^ (Figure 2); this finding illustrates that placing virtually all substituents that have electron‐withdrawing ability in a chelating benzylidene ligand can activate the Hoveyda–Grubbs catalyst.

We reasoned that the role of the electron‐withdrawing group (EWG) is to decrease the electron density on the oxygen atom of the chelating *i*PrO fragment, thus weakening the strength of the Ru−O bond (Figure 2, insert).^[21]^ This makes the corresponding catalysts activate faster and prevent them from entering into an inactive “sleeping” state through a so‐called “boomerang” mechanism.[^18^, ^29^] In an independent study, Blechert and co‐workers reported on a set of Hoveyda–Grubbs catalysts substituted with electron‐donating groups (EDGs, mostly ethers; deactivating) and EWGs (F, CF_3_, CN; activating) and interpreted differences in their activities with the aid of σ Hammett constants (Table 1).^[30]^ Butenschön and co‐workers disclosed an interesting and highly active bimetallic catalyst (**Ru13**), where a Cr(CO)_3_ fragment in the π‐arene complex was used to induce both steric and electronic (EWG) activation of the parent Hoveyda–Grubbs catalyst (Figure 2).^[31]^

**Table 1:** Comparison of Hammett constants (*σ_para_*) of various functional groups.^[35]^

Group	*σ_para_*	Group	*σ_para_*
F	0.06	C(O)C_6_H_5_	0.43
CF_3_	0.54	SO_2_N(CH_3_)_2_	0.65
NO_2_	0.78	NHSO_2_CF_3_	0.39
SO_2_C_6_H_5_	0.68	NHC(O)CF_3_	0.12

Based on the above‐described EWG‐activating effect, more catalysts have been logically developed and eventually commercialized, including those bearing SO_2_NR_2_,^[32]^ NHCOR^[33]^ (Figure 3), and PO(OR)Ar^[34]^ EWGs.

The nitro catalyst **Ru12** ^[36]^ and Mauduit's activated catalysts,^[37]^ such as **Ru15** and **Ru16**, have been reviewed previously and, therefore, in the present Review only new facts and the most instructive examples of their use will be provided. To the best our best knowledge, the third most popular EWG analogue (EWG=SO_2_NR_2_), the Zhan‐1B catalyst **Ru14**, is included in a review for the first time.

## 3 Synthesis of Advanced Biologically Active and Natural Compounds by Utilizing EWG‐Activated Catalysts

### 3.1 Examples in Ring‐Closing Metathesis

**Scheme 2:**
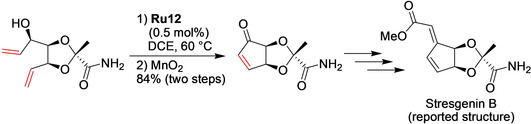
One pot RCM and oxidation in the total synthesis of the reported structure of Stresgenin B. DCE=1,2‐dichloroethane.

Total synthesis sometimes allows a conclusion to be made that a reported structure of a given natural product requires revision.[^38^, ^39^, ^40^] This was the case in the recent disclosure from Chan and Koide on the first total synthesis of the reported structure of the heat shock protein expression inhibitor Stresgenin B (Scheme 2).^[41]^ The synthesis features a number of synthetically challenging transformations, and among them the one‐pot^[42]^ ring‐closing metathesis/oxidation event using a properly chosen, not‐too‐high amount of nitro catalyst **Ru12**, and then MnO_2_ as the oxidant ensured a high yield (84 %) of the required intermediate. This is a nice example of a well‐planned and skillfully executed but rather straightforward ring‐closure metathesis (RCM) reaction.

**Scheme 3:**
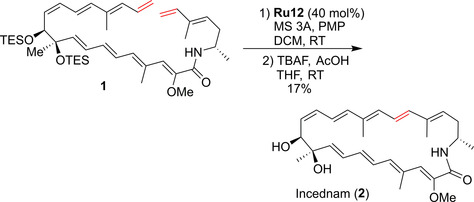
RCM macrocyclization step in the synthesis of Incednam.^[43]^ PMP=*p*‐methoxyphenol; TES=triethylsilyl.

The group from Keio University reported an improved total synthesis of incednam (**2**), the aglycon of the 24‐membered macrolactam glycoside antibiotic Incednine.^[43]^ The retrosynthetic analysis of **2** was based on the construction of the 24‐membered macrocycle by a challenging intramolecular macrocyclization of the fragile polyunsaturated substrate **1** (Scheme 3). Conditions for the RCM were rigorously explored, such as screening of a number of catalysts, including first‐ and second‐generation Grubbs and Hoveyda–Grubbs catalysts, as well as nitro catalyst **Ru12**. These tests revealed that the best conditions consist of using **Ru12** in the presence of *p*‐methoxyphenol (PMP)^[44]^ and 3 Å molecular sieves. Despite such measures, Incednam (**2**) was obtained in only 17 % overall yield after cleavage of the TES groups (Scheme 3).

**Scheme 4:**
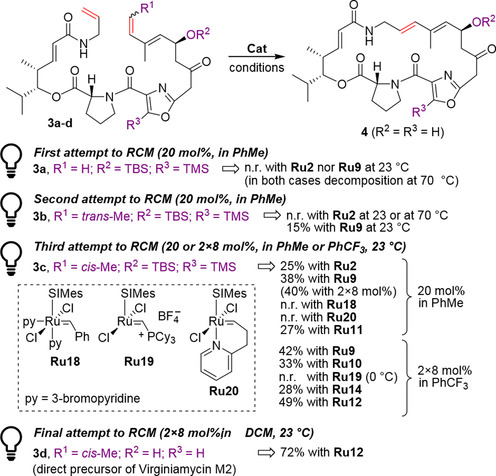
Detailed optimization of the cross‐metathesis step in the synthesis of Virginiamycin M2 (**4**). n.r.=no reaction; py=3‐bromopyridine; TBS=*tert*‐butyldimethylsilyl; TMS=trimethylsilyl.

The next example shows another challenging RCM reaction, where the laborious optimization of the substrate structure, as well as a wise selection of the catalysts and conditions changed the initial failure into a success. Researchers from the University of California in San Francisco reported the total synthesis of Virginiamycin M2, a member of the streptogramin natural product group.^[45]^ The retrosynthesis featured a late‐stage macrocyclization to form the 23‐membered ring of Virginiamycin (Scheme 4). Different cyclization reactions were tried, and after the failure of a Pd‐catalyzed Stille coupling, the authors turned to Ru‐catalyzed olefin metathesis. The initial efforts were rather disappointing, as the reactions of bis‐terminal precursor **3 a** with first‐ and second‐generation Grubbs and Hoveyda–Grubbs catalysts at 23 °C resulted in no conversion, whereas a higher temperature (70 °C) resulted in the substrate being consumed but no cyclic product detected (probably because of the presence of the rather fragile conjugated diene fragment). Thus, the authors resorted to alteration of the substrate structure. The *trans*‐methyl‐substituted precursor **3 b** still led to unsatisfactory results with Grubbs catalysts, but the expected macrocycle was finally observed in the reaction mixture when the more robust second‐generation Hoveyda–Grubbs catalyst (**Ru9**) was used. Although the yield of only 15 % had little practical utility, this result showed that the RCM macrocyclization is indeed possible, and just required more detailed optimization. Therefore, the authors switched to the *cis*‐isomer **3 c** and tested a wider set of metathesis catalysts. Although most of them (such as the polymerization catalysts) did not improve the yield of the target macrocycle, it was found that a batch‐wise addition of a catalyst (2×8 instead of 20 mol % added in one portion) and a more polar solvent (PhCF_3_ instead of PhCH_3_) led to small improvements.[^46^, ^47^] Under these conditions, the best catalysts were EWG‐activated **Ru14** and **Ru12**, which provided the expected macrocycle in yields of 28 and 49 %, respectively. Interestingly, when the desilylated substrate (**3 d**) was used in the RCM reaction instead of **3 c**, the productivity of the key macrocyclization reaction increased greatly, with Virginiamycin M2 (**4**) isolated in 72 % yield when using **Ru12** in CH_2_Cl_2_ at room temperature (Scheme 4).^[45]^

Although the cause of this improvement is unclear, the authors suggested that it can arise from the coordination of the catalyst to the exposed allylic alcohol or from favorable conformational bias in the “unprotected” macrocyclic precursor **3 d** relative to **3 c**.^[45]^ Whatever the reason for this fortuitous effect is, the reported study shows the importance of a careful optimization of the metathesis step, both by adjustments made in the substrate structure and by the right choice of the catalyst. This fact can be seen in numerous examples in the literature,^[3]^ and the proficient cooperation of organic synthetic chemists with experts in metathesis appears to be the easiest way to optimize such challenging metathesis processes.

**Scheme 5:**
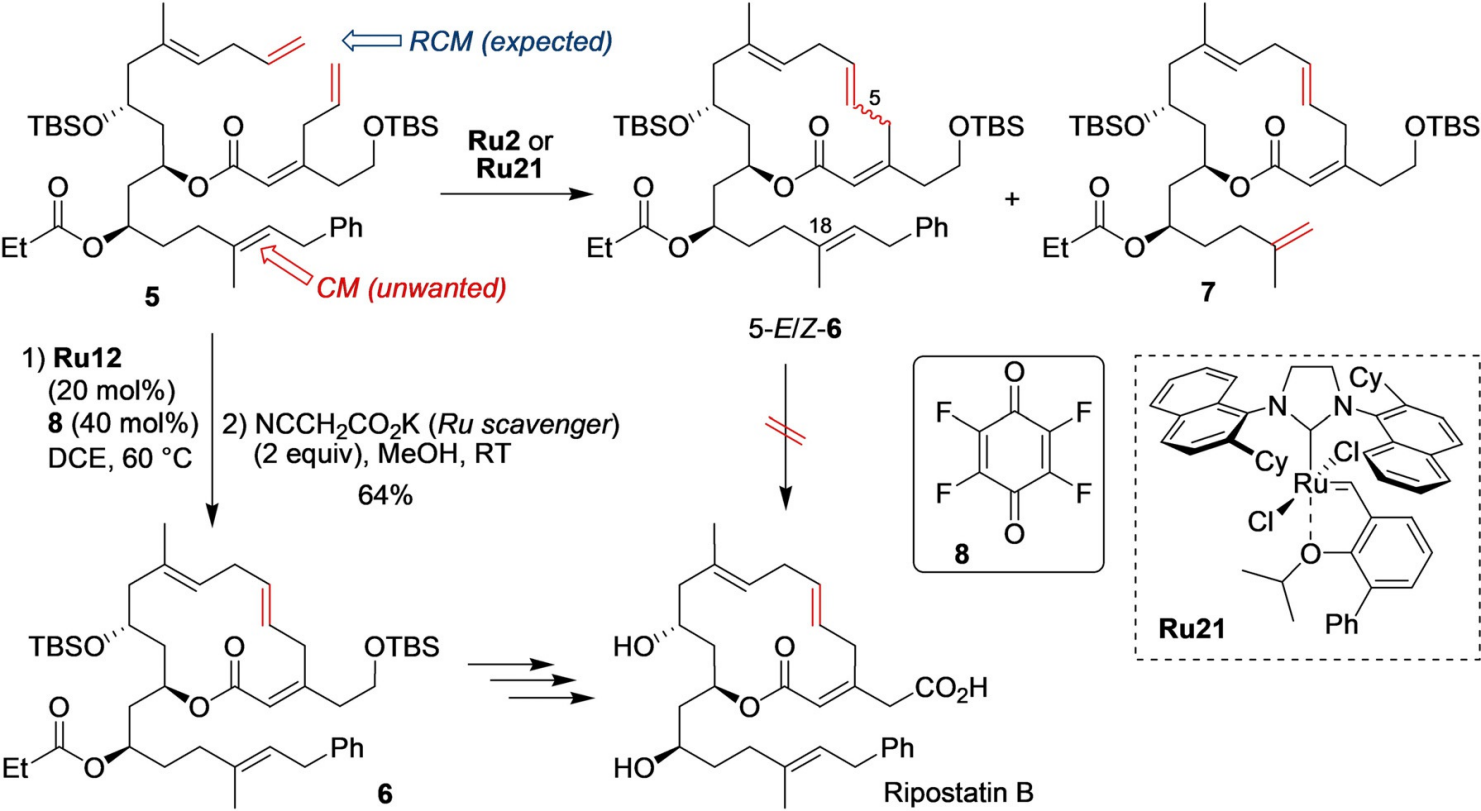
Problematic RCM in the total synthesis of Ripostatin B. Cy=cyclohexyl.

Another example where the key RCM step is a real challenge was reported by Christmann and co‐workers in the total synthesis of the RNA polymerase inhibitor Ripostatin B.^[48]^ The authors planned to construct the sensitive 14‐membered macrolactone, which features the peculiar doubly skipped triene, by means of RCM. Unfortunately, a number of problems were encountered with popular catalysts, such as first‐ and second‐generation Grubbs or Hoveyda–Grubbs **Ru9**, including loss of the *E*/*Z* selectivity, sluggish reactions, or olefin truncation (to form **7**; Scheme 5). The last “parasitic process”, caused by cross‐metathesis with ethylene produced during the reaction, strongly depended on the nature of the catalyst. Whereas the **Ru2** catalyst (20 mol %) led to two products (both of them useless for obtaining the target molecule)—*E*/*Z*‐**6** and truncated **7**—in an approximately 1:1 ratio, the Dorta catalyst^[49]^ **Ru21** (10 mol %) showed a perverse selectivity, giving **7** as the major product (but as a single *E* isomer!). Careful analysis of these failures led to an improvement. Using catalyst **Ru12** together with tetrafluoro‐1,4‐benzoquinone (**8**) known for its anti‐isomerization properties,^[50]^ and purging with argon minimized the problems encountered previously and allowed the expected product **6** to be obtained in an acceptable yield and with full *E*‐selectivity in the C−C double‐bond formation (Scheme 5).^[48]^ Interestingly, the authors used an isocyanide reagent^[51]^ to quench the Ru metathesis catalyst just after the RCM was completed, thus preventing unwanted metathesis side reactions.[^52^, ^53^, ^54^]

**Scheme 6:**
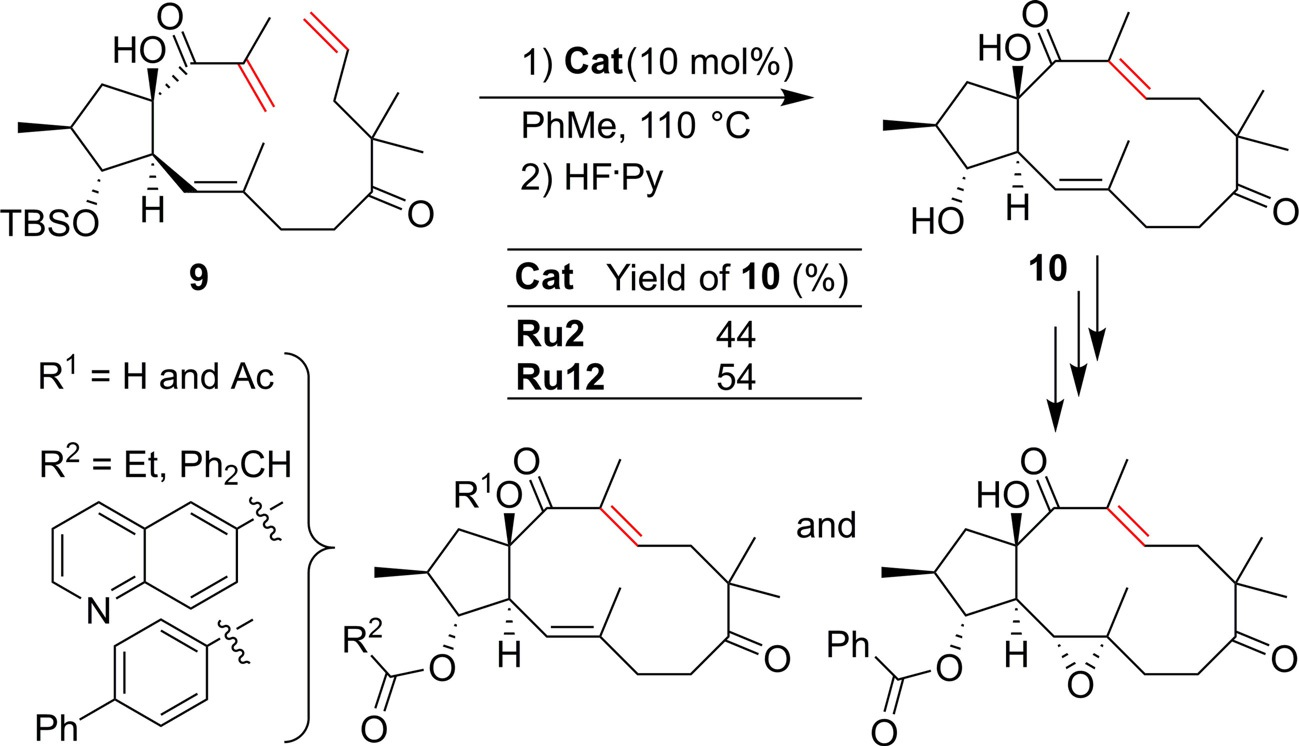
Synthesis of natural and non‐natural Jatrophane diterpenes. Ac=acetyl.

Wiese and Hiersemann reported another challenging RCM macrocyclization in the synthesis of natural and non‐natural Jatropha‐5,12‐dienes.^[55]^ The regioselective RCM reaction of triene **9** was expected to establish the fully substituted 12‐membered *trans*‐bicyclo[10.3.0]pentadecane framework of the target compounds. Unfortunately, in the initial trials it was found that the first‐generation Grubbs catalyst led to no conversion and the second‐generation Hoveyda–Grubbs catalyst delivered a low (22 %) yield of the expected 3‐*epi*‐characiol **10**. To the authors relief, the crucial RCM could then be realized using catalysts **Ru2** and **Ru12**, which afforded, after removal of the remaining protecting groups, the expected key product **10**, which was later transformed into a number of natural and non‐natural members of the Jatrophane family of diterpenes (Scheme 6).[^55^, ^56^]

**Scheme 7:**
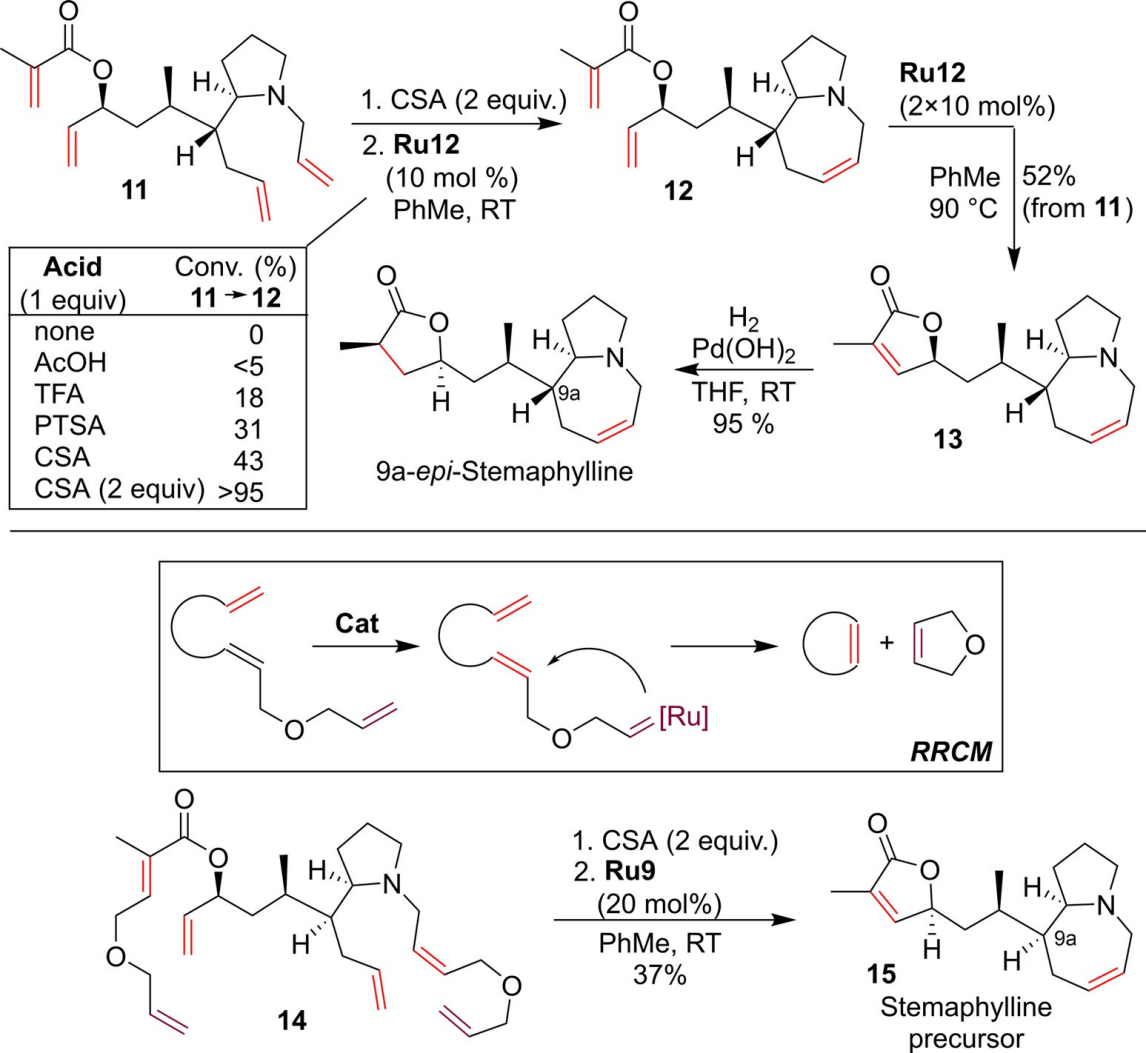
Problematic RCM in the synthesis of 9a‐*epi*‐Stemaphylline and Stemaphylline. TFA=trifluoroacetic acid; CSA=camphorsulfonic acid; PTSA=*p*‐toluenesulfonic acid.

The metathesis of substrates containing basic nitrogen atoms (e.g. alkaloids precursors) are known to be difficult and require the use of acid additives to prevent coordination of the amine to the ruthenium center, which can result in catalyst poisoning. Researchers from the Vanderbilt Center for Neuroscience Drug Discovery reported a very interesting total synthesis of the *Stemona* alkaloid Stemaphylline, its C9a‐epimer, and their *N*‐oxides.^[57]^ Ambitiously, it was planned to close both the 5‐ and 7‐membered rings of these natural products in two independent one‐pot RCM events (Scheme 7). Unfortunately, the conditions (**Ru2**, trifluoroacetic acid) developed for other azabicyclic ring systems^[58]^ proved to be unsuccessful in the case of tetraene **11**. Fortunately, **Ru12** in the presence of the same acid (1 equiv) gave the desired product **12**, albeit in low yield. Interestingly, this challenging RCM reaction was found to be sensitive to the nature and amount of the acid used to protonate the basic nitrogen atom present in **11**. The best results were achieved with two equivalents of camphorsulfonic acid (CSA). Using optimized conditions for closure of the azepine ring, the authors tried the far more ambitious tandem bis‐ring closure to obtain bis‐RCM product **13** in one reaction. A small selection of metathesis catalysts were screened again (Schrock molybdenum catalyst and a number of Ru catalysts: **Ru2**, **Ru9**, **Ru12**, **Ru14**). From this set, complexes **Ru9** and **Ru12** were found to be optimal, and finally the reaction of tetraene **11** in the presence of CSA was conducted with **Ru12** to give **13** in 52 % yield (from **11**). This product was then converted in high yield into 9a‐*epi*‐Stemaphylline (Scheme 7) and 9a‐*epi*‐Stemaphylline *N*‐oxide.^[57]^

Encouraged by this success, the researchers approached the synthesis of Stemaphylline, an alkaloid which differs from 9a‐*epi*‐Stemaphylline in the configuration at just one stereocenter.[^59^, ^60^] Surprisingly, this relatively small change made the analogous RCM reaction fail. The authors put a lot of effort into understanding the reasons for such a large difference in reactivity between the 9a‐*epi*‐Stemaphylline and Stemaphylline tetraene precursors, and finally opted to use the relay ring‐closing‐metathesis (RRCM) strategy to save the project.^[61]^ RRCM is an important technique that was developed to force some recalcitrant substrates to enter the metathesis cycle or to differentiate between competitive metathesis pathways.[^61^, ^62^] Typically, it can be carried out by introducing a special relay‐arm into a substrate molecule, to which the Ru‐carbene moiety can attach more easily, and then undergoing a sequence of intramolecular transformations (including the release of a stable cyclic by‐product) to yield, at the end, the desired cyclic olefin (see insert in Scheme 7). Luckily, the application of the RRCM method to the specially designed substrate **14** led (after additional optimization steps) to formation of the desired Stemaphylline precursor **15**, although in moderate yield (37 %; Scheme 7, bottom).

**Scheme 8:**
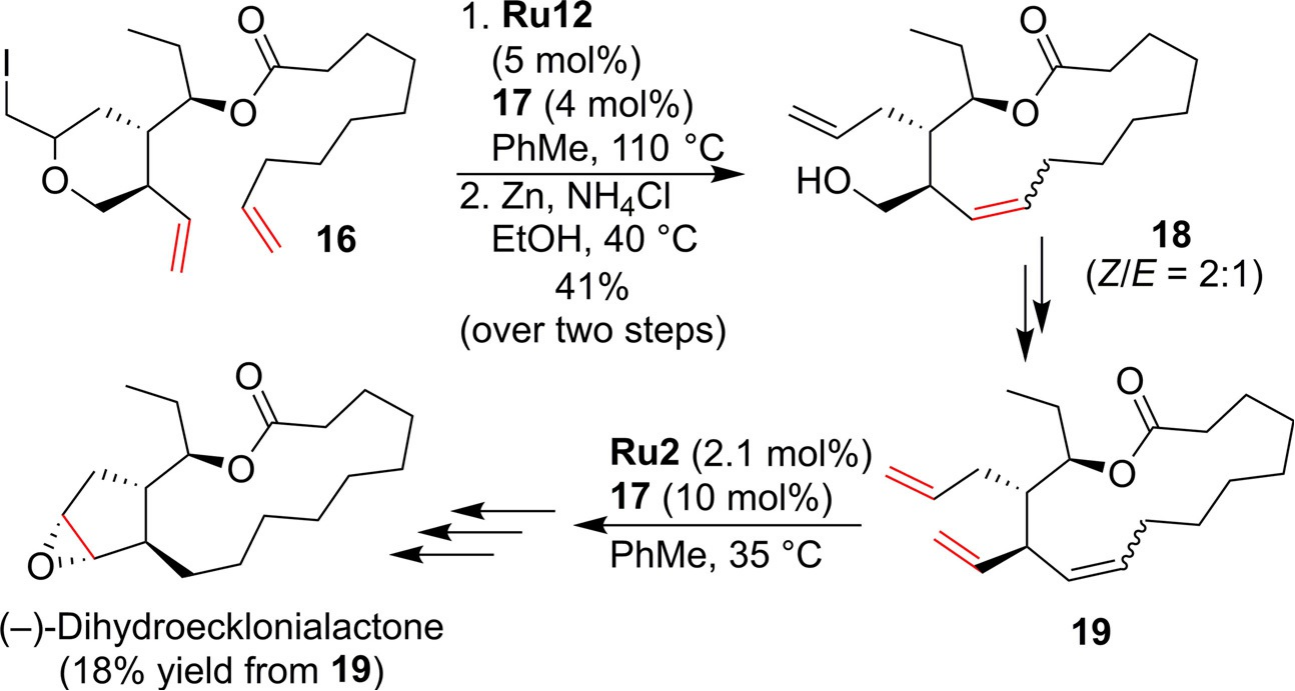
RCM in the synthesis of (−)‐Dihydroecklonialactone B.

Hiersemann and co‐workers reported an elegantly designed total synthesis, where RCM was used to close both the 5‐ and the 14‐membered rings of (−)‐9,10‐Dihydroecklonialactone B.^[63]^ The envisioned formation of the 14‐membered lactone seemed rather straightforward (RCM of a rather simple bis‐terminal diene); unfortunately, this endeavor turned out to be much more challenging than expected. After screening a number of catalysts and conditions, the nitro catalyst **Ru12** (5 mol %) in the presence of 1,4‐benzoquinone (**17**; 0.1 equiv) at elevated temperature was found to perform best (Scheme 8). To avoid decomposition, this rather unstable intermediate was immediately subjected to Zn‐mediated β‐elimination to deliver the lactone **18** in 41 % yield (after two steps). This was transformed into triene **19** for the second RCM event. The second RCM (this time using catalyst **Ru2** to promote the cyclopentene formation) provided the bicyclic core of the target molecule. Further standard transformations provided access to (−)‐9,10‐Dihydroecklonialactone B (Scheme 8).

The examples outlined above, and those that had to be skipped because of limited space,[^64^, ^65^, ^66^, ^67^, ^68^, ^69^, ^70^, ^71^] testify the importance of the detailed optimization of a RCM reaction, but also shows that it is sometimes rather difficult to rationalize why two seemingly very similar catalysts or substrates give substantially different results.^[72]^

### 3.2 Examples in Cross‐Metathesis Reactions

Cross‐metathesis reactions were for a long time considered as technically more difficult than ring‐closing metathesis.^[73]^ With the introduction of modern olefin metathesis catalysts, cross‐metathesis reactions with α,β‐unsaturated compounds, such as acrylic esters, acrolein, or vinyl ketones became possible and now are frequently used in target‐oriented syntheses. The advantage of this approach is that the two reacting partners exhibit different character and reactivity. In particular, the less‐reactive electron‐deficient olefin typically does not enter into parasitic self‐metathesis reactions (Scheme 1), and can be used in excess, thus allowing for high selectivity and a high yield with cross‐metathesis. From the numerous Ru catalysts available, EWG‐activated complexes such as **Ru12** or **Ru14** usually give good results.[^74^, ^75^, ^76^, ^77^]

**Scheme 9:**
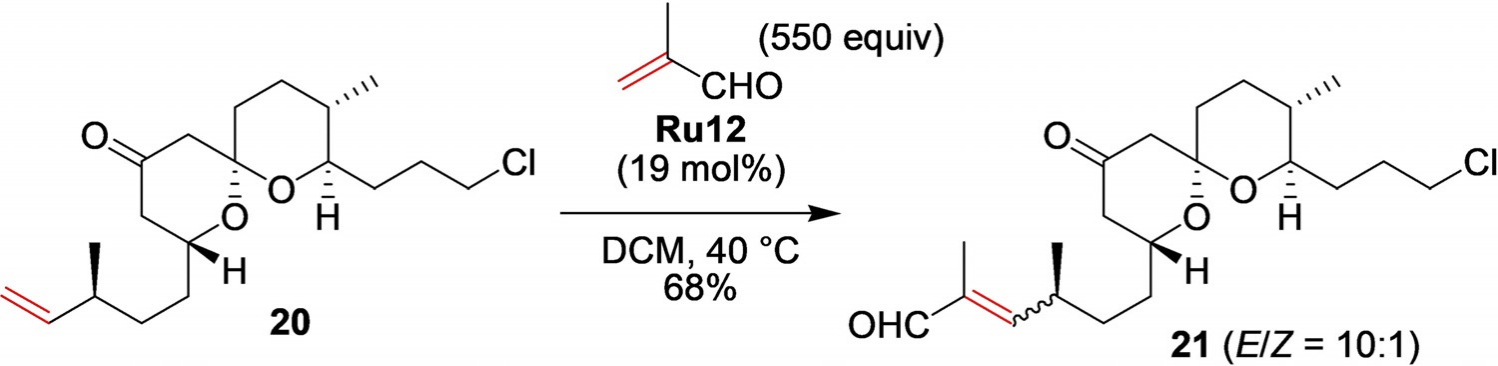
Cross‐metathesis with a large excess of metacrolein in the synthesis of an intermediate to Bistramide A analogues.

Less‐active substituted α,β‐unsaturated compounds, such as methacrolein, can also be utilized in cross‐metathesis. Researchers from the University of Pittsburgh reported the synthesis of unnatural Bistramide A analogues, with the purpose of comparing their potency with that of the natural product.^[78]^ The RCM step consisted of a reaction between spirocycle **20** and 550 equivalents methacrolein in the presence of nitro catalyst **Ru12**, which led to the product **21** in an acceptable yield (Scheme 9). This universal intermediate was then used to obtain a number of Bistramide A analogues.^[78]^

**Scheme 10:**
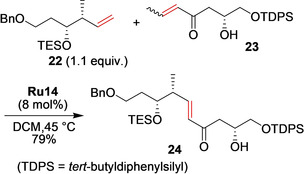
Synthesis of Mandelalide A precursor **24** by cross‐metathesis.

Fürstner and co‐workers reported the concise synthesis of the putative structure of the highly cytotoxic marine macrolide Mandelalide A.[^79^, ^80^] Cross‐metathesis between terminal alkene **22** and functionalized enone **23** worked very well in the presence of **Ru14** and furnished the required enone building block **24** with high *E*‐selectivity (Scheme 10).

**Scheme 11:**
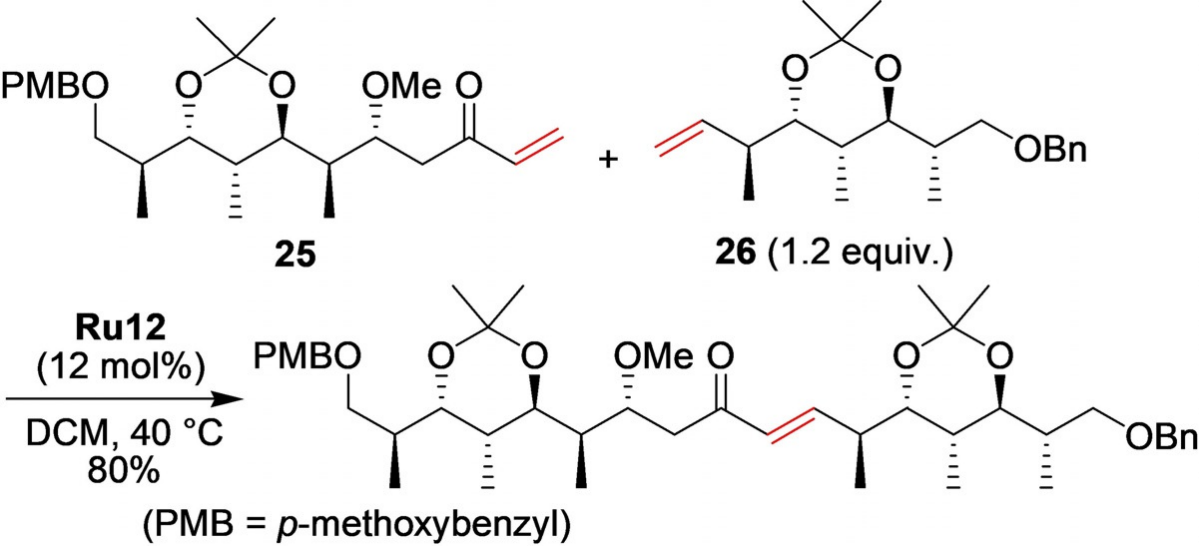
Synthesis of a precursor of the cytotoxic spiroketal Spirangien A by cross‐metathesis.

Similarly, the successful cross‐metathesis between advanced enone building block **25** and functionalized alkene **26** mediated by nitro‐activated **Ru12** was reported in the synthesis of the cytotoxic spiroketal Spirangien A (Scheme 11).^[81]^

The case where two reacting olefins are of similar reactivity is more complicated, as each partner can independently undergo “dimerization” through homometathesis (Scheme 1), thereby limiting the yield of the key cross‐metathesis reaction, and giving a mixture of products that is often complicated to separate. The following examples show how to deal with such a problem.

**Scheme 12:**
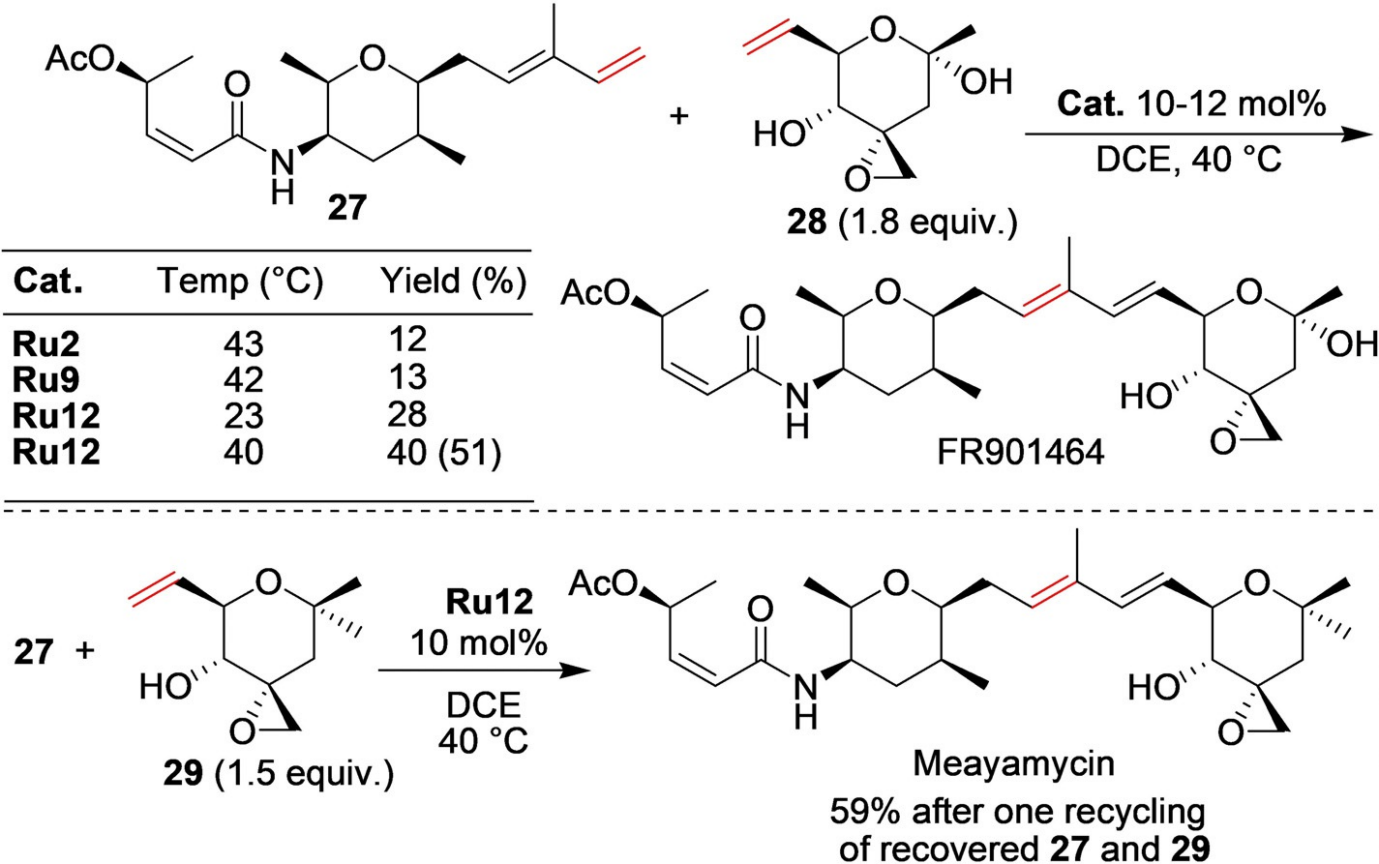
Cross‐metathesis as the last step in the synthesis of FR901464 and Meayamycin. The value in parenthesis shows the yield based on recovered **27**.

The structurally unique FR901 464 was isolated at the Fujisawa Pharmaceutical Company from the culture broth of a bacterium of Pseudomonas species, and proven to possess antitumor activity against a number of cell lines, thus having the potential for clinical application.^[82]^ Koide and co‐workers targeted the synthesis of FR901464 and their analogues.^[83]^ After the failure of a number of synthetic strategies, in the final attempt, Koide and co‐workers envisaged forming the key C−C double bond by cross‐metathesis as the very last step in the synthesis.^[83]^ As shown in Scheme 12, cross‐metathesis between **27** and **28** was not a trivial task, and the proper choice of the catalyst was the key. The fragile nature of **28** prevented using more forcing reaction conditions, because the reacting partners quickly decompose above 47 °C. Therefore, the conditions identified by Koide and co‐workers involved the use of 12 mol % **Ru12** in 1,2‐dichloroethane (DCE) at precisely 40 °C. The cross‐metathesis partner **28** was used in an excess of 1.8 equivalents to maximize the consumption of the other partner (**27**). This allowed FR901464 to be obtained in 40 % yield after one recycle of the unreacted starting materials (51 % yield based on recovered **27**). Encouraged by the success of the above strategy, Koide and co‐workers decided to synthesize the FR901464 analogue Meayamycin, which was achieved in 59 % yield by the same strategy after one recycle of the recovered starting materials (Scheme 12).^[83]^ Later, more than a dozen other analogues of FR901464 were prepared by using similar **Ru12**‐catalyzed late‐stage cross‐metathesis reactions, and some of them have been shown to be significantly more potent than Meayamycin against several cancer cell lines and, therefore, of interest in oncology.^[84]^

**Scheme 13:**
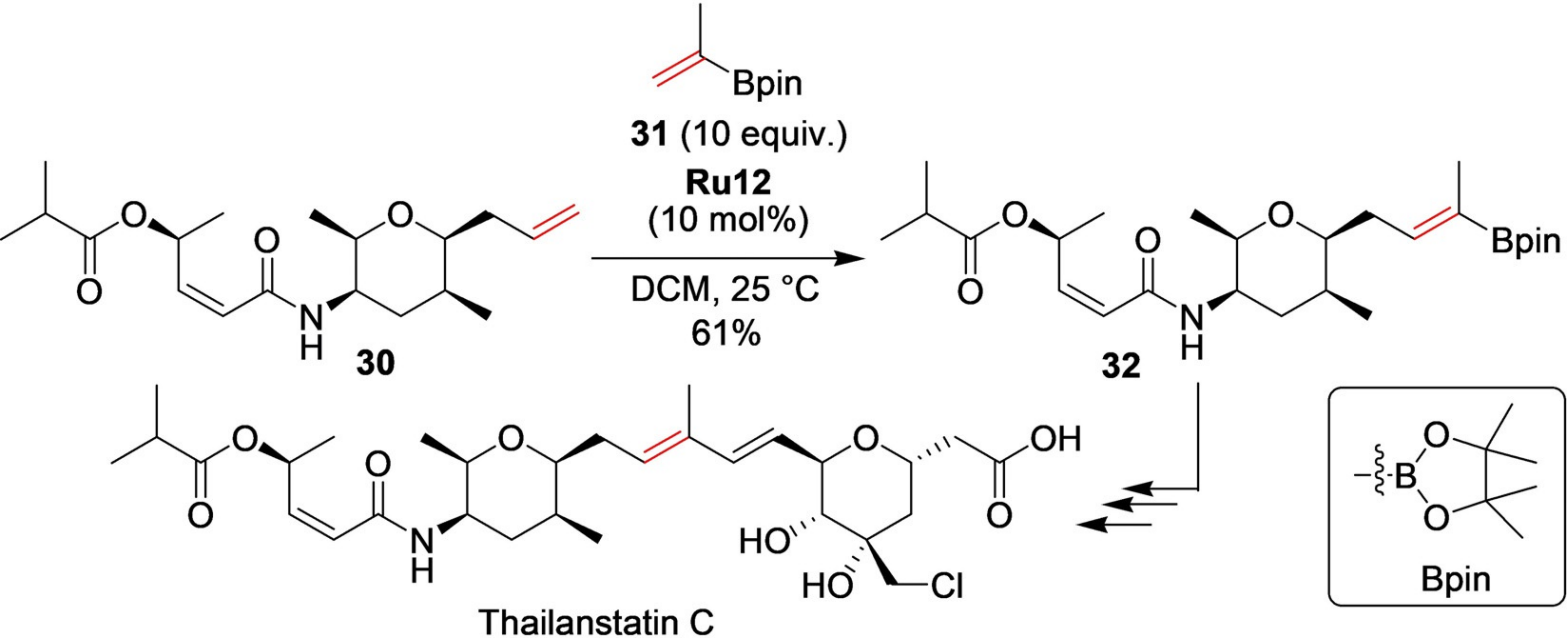
Cross‐metathesis during the total synthesis of Thailanstatin C.

Nicolaou et al. reported the efficient and selective total syntheses of natural products exhibiting a related structure—Thailanstatins A–C.^[85]^ En route to these advanced targets, the full orchestra of transition‐metal‐catalyzed transformations—including a number of olefin metathesis events—was used but, unlike in Koide's retrosynthesis, the key step to combine the two advanced fragments of the natural product was a Suzuki coupling reaction, not metathesis. The boronate precursor (**32**) for the key Suzuki step was, however, made by a **Ru12**‐catalyzed cross‐metathesis with vinyl boronate **31** (Scheme 13). This successful cross‐metathesis of the rather challenging boronate partner **31** was then repeated multiple times in the synthesis of numerous analogues of Thailanstatin, thereby allowing for evaluation of their cytotoxicity against a number of cancer cell lines.^[85]^

Cross‐metathesis with vinyl borolanes is a popular maneuver in target‐oriented syntheses.^[73]^ Another example is the conversion of the chiral homoallylic alcohol (PMB‐protected) **33** into *trans*‐vinylborolane **34** in high yield and stereoselectivity (*E*/*Z*>20:1; Scheme 14).^[86]^ A number of cross‐metathesis events between vinyl boronates and advanced olefinic building blocks catalyzed by **Ru2** and **Ru12** have also been used in the synthesis of Rhizopodin.^[87]^

**Scheme 14:**
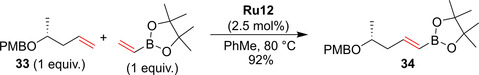
Cross‐metathesis of vinyl borolane in the synthesis of Rhizopodin.

**Scheme 15:**
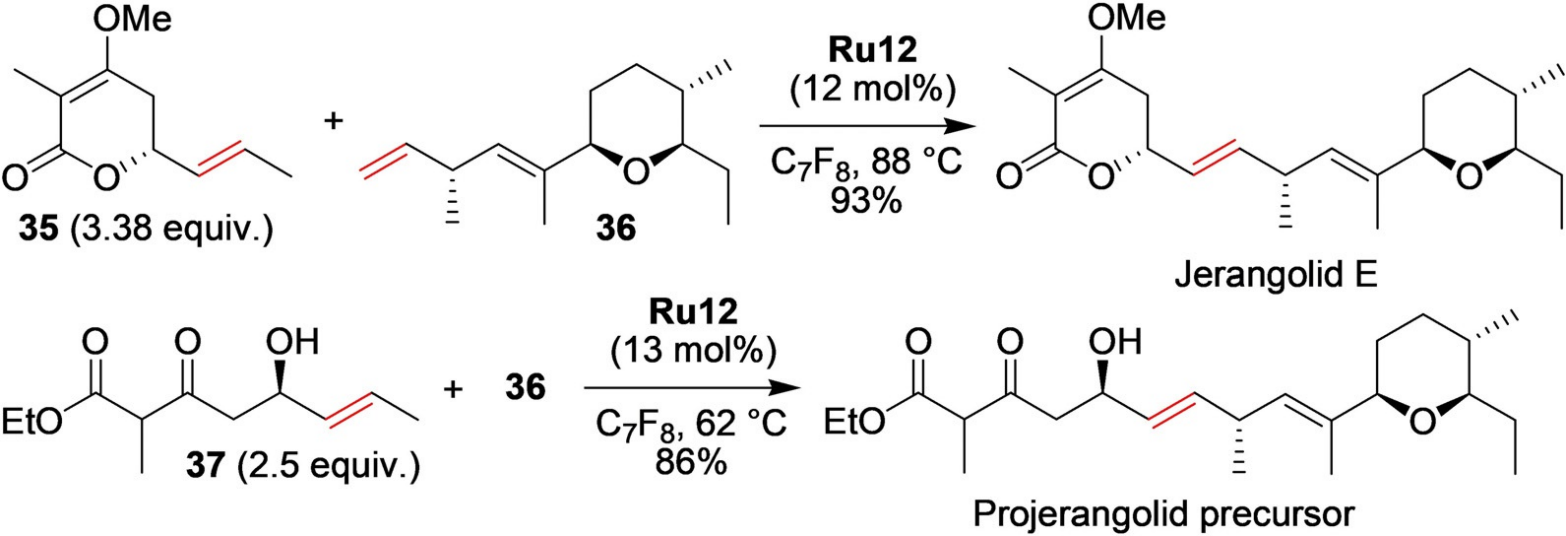
Synthesis of Jerangolid E and the Projerangolid precursor. C_7_F_8_=perfluorotoluene.

Another impressive example where two complex alkene fragments are combined to form a target molecule in the last step has been presented by Hahn and co‐workers in the total synthesis of Projerangolid and Jerangolid E.^[88]^ Reacting an excess of the less‐reactive olefin **35** with diene **36** in the presence of the second‐generation Grubbs catalyst led to the isolation of Jerangolid E in 23 % yield. In contrast, using **Ru12** in perfluorinated toluene[^46^, ^89^, ^90^] delivered the target compound in 93 % yield and excellent *E*‐selectivity (Scheme 15). The established cross‐metathesis conditions were then applied for the synthesis of Projerangolid, as well as for non‐natural 5‐*epi*‐Projerangolid and 9‐(*Z*)‐Jerangolid E.^[88]^

**Scheme 16:**
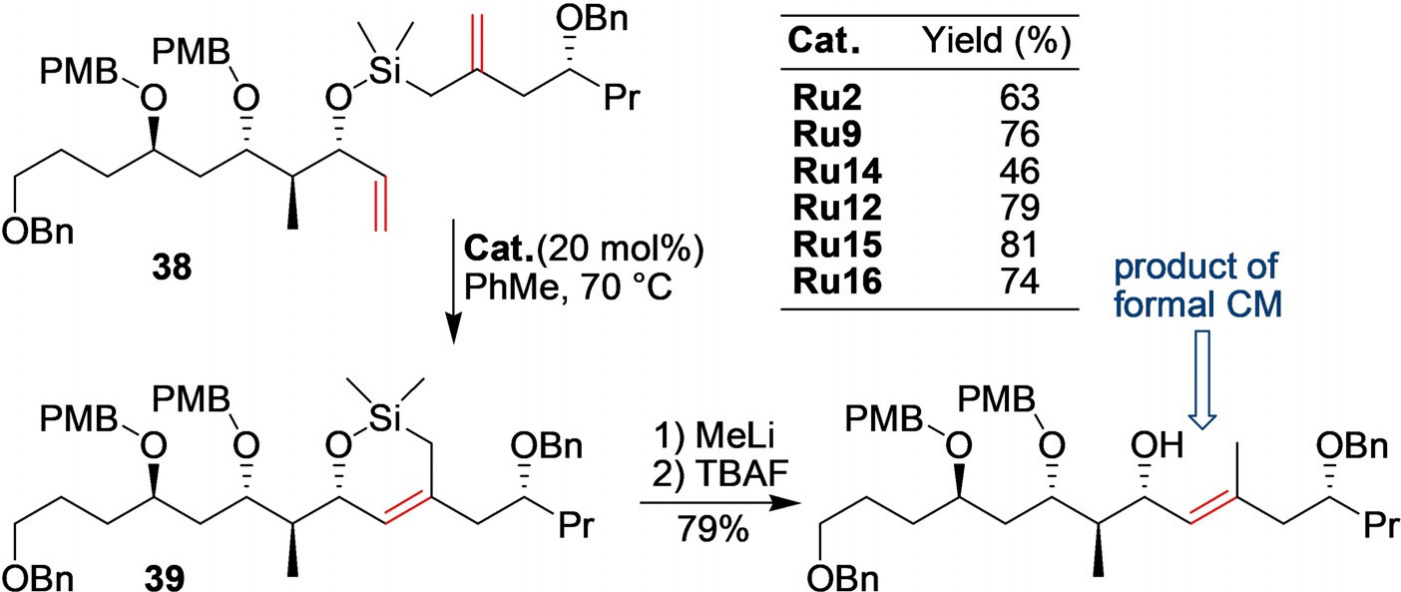
Synthesis of the C16–C30 fragment of Dolabelide C by using a silicon tether strategy. PMB=*p*‐methoxybenzyl.

When the direct cross‐metathesis reaction is for some reasons impossible or not selective enough, RCM of a temporary tether can lead to better results. Prunet and co‐workers tried this strategy recently.^[91]^ During the previous studies toward the synthesis of Dolabelide C, the C16–C30 fragment (Scheme 16) of this natural product was obtained using a cross‐metathesis reaction.^[92]^ Unfortunately, despite a large amount (45 mol %) of the Hoveyda–Grubbs catalyst having been used, the cross‐metathesis reaction yield was only 47 %, thus showing the limitations of cross‐metathesis for the synthesis of highly hindered trisubstituted olefins. In the more recent approach, Prunet and co‐workers used a silicon‐tether RCM strategy to obtain the same C16–C30 fragment of Dolabelide C. An interesting catalyst influence was noted in this RCM reaction (Scheme 16). Whereas second‐generation Grubbs catalysts afforded an already satisfactory yield of 63 % (compared with the previous cross‐metathesis‐based synthesis), the Hoveyda–Grubbs catalyst improved it to 76 %. The Zhan‐1B catalyst was the least productive, while the nitro and the Mauduit catalysts were superior, with an impressive 81 % yield of **39** obtained with the Umicore M71 SIMes complex.^[91]^

There are more examples of cross‐metathesis that are worth discussing,[^74^, ^75^, ^76^, ^77^, ^93^, ^94^, ^95^, ^96^] but the limited space of this Review does not allow this.

### 3.3 Examples in Enyne Cycloisomerization

**Scheme 17:**
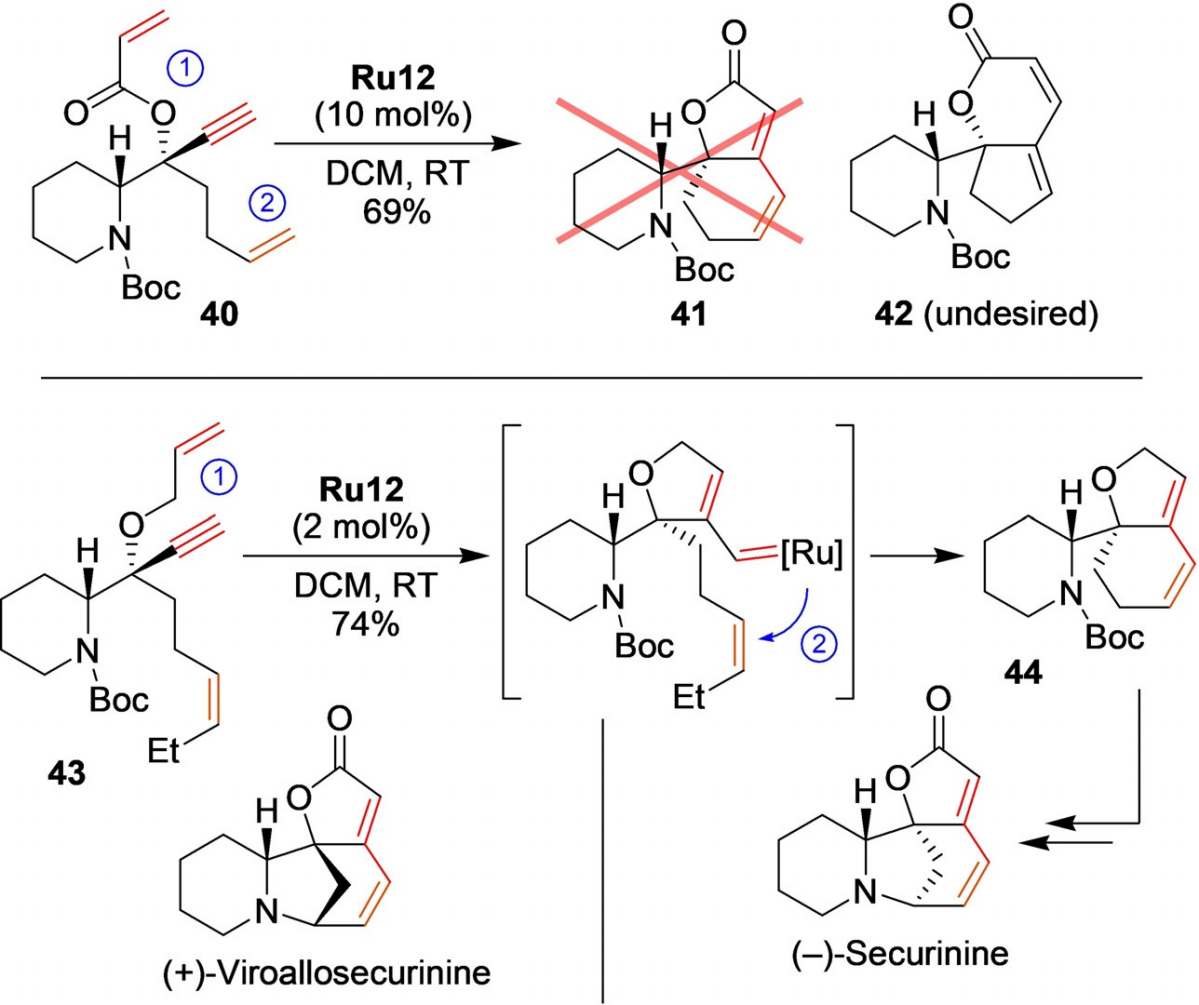
Solving the riddle in the synthesis of Securinega alkaloids. Boc=*tert*‐butyloxycarbonyl.

One of the many nice examples where enyne cycloisomerization was utilized in the synthesis of advanced natural products is Honda's long‐term study on Securinega alkaloids.^[97]^ These naturally occurring alkaloids exhibit attractive biological activities and constitute an ambitious synthetic target. In one of the retrosynthetic approaches, Honda et al. intended to employ a tandem one‐pot enyne cycloisomerization followed by a ring‐closing metathesis (enyne‐RCM) of enyne acrylic ester **40** as the key reaction (Scheme 17, top). However, the treatment of this substrate with **Ru12** afforded the δ‐lactonic compound (**42**) instead of the desired compound **41**. Clearly, a ruthenium carbene generated from the terminal alkene in the butenyl group reacted with the alkyne prior to the C−C double bond of the α,β‐unsaturated ester (②‐then‐① instead of ①‐then‐②, see Scheme 17 top). To solve the problem, a less‐reactive alkene moiety (bearing a sacrificial ethyl end group) and a more reactive allyl ether (instead of the acrylic ester) were introduced in the modified substrate **43**. In this case, the tandem enyne‐RCM reaction proceeded very well with only 2 mol % of the highly active catalyst **Ru12** and gave the properly cyclized product (**44**) in 74 % yield (Scheme 17, bottom). Thus, the stereoselective construction of the remaining rings was achieved in a relatively short sequence, completing the first synthesis of enantiomerically pure (−)‐Securinine.^[98]^ A similar sequence was then used to obtain (+)‐Viroallosecurinine.^[99]^ The above enyne cycloisomerization followed by RCM sequence was later modified in a very clever way by Yang, Li, and co‐workers to obtain other Securinega alkaloids: (−)‐Norsecurinine, (+)‐Allonorsecurinine, (−)‐Flueggine A, and (+)‐Virosaine B.^[100]^ The key enyne cycloisomerization followed by RCM reactions were tested using several commercially available metathesis catalysts, including second‐generation Grubbs and Hoveyda–Grubbs catalysts, but the EWG‐activated Zhan‐1B catalyst (**Ru14**) provided the best results.^[100]^

## 4 Applications in the Synthesis of Active Pharmaceutic Ingredients

Applications of olefin metathesis in the preparation of active pharmaceutic ingredients (APIs) have recently been reviewed.[^17^, ^101^, ^102^, ^103^] Therefore, only a few of examples will be described herewith, as they nicely show current challenges related to the use of metathesis technology in the pharmaceutical industry.

**Figure 4:**
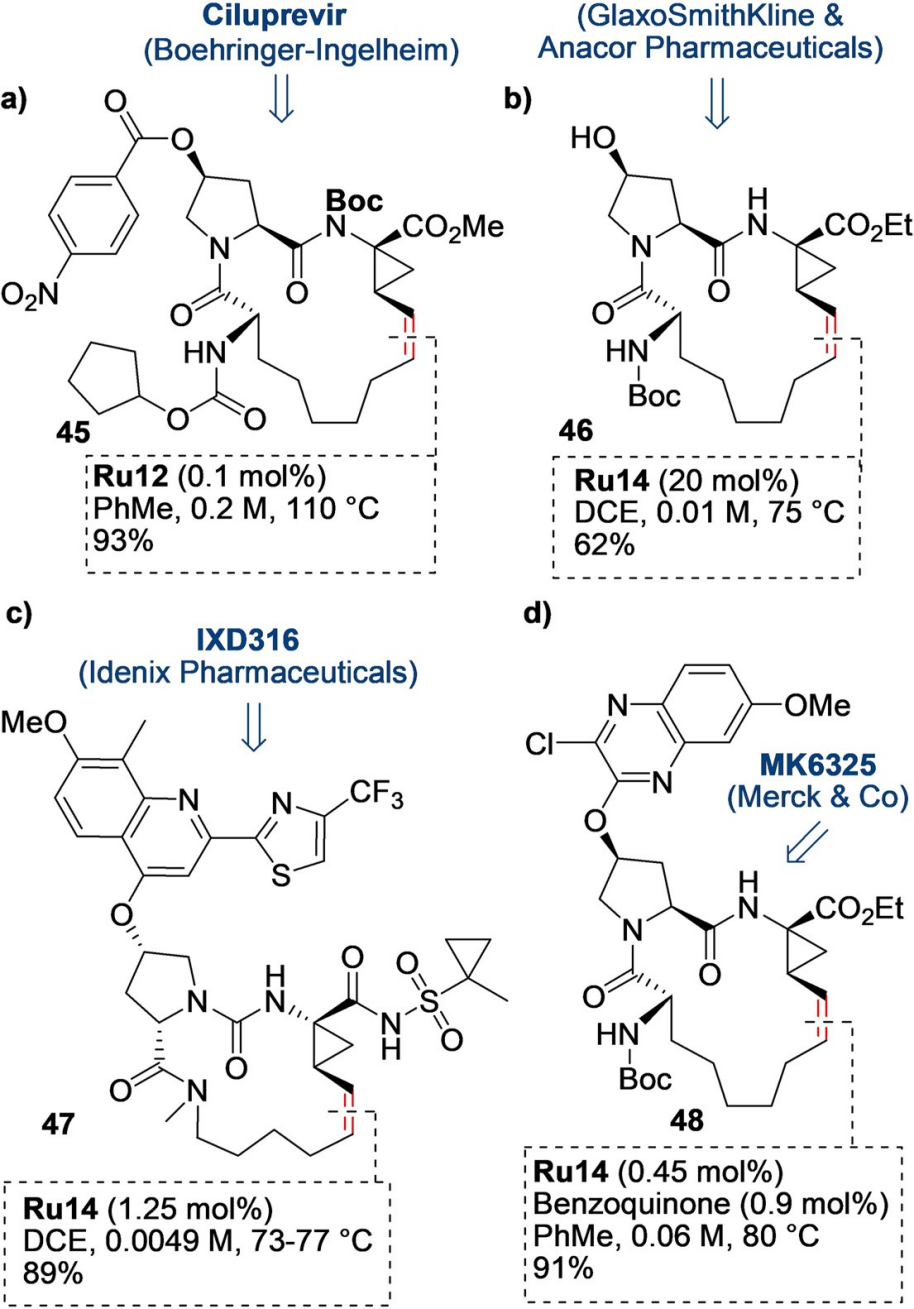
Macrocyclic precursors of four selected APIs obtained by RCM.

The hepatitis C virus (HCV) is a major cause of chronic liver disease and can lead to cirrhosis, carcinoma, and liver failure. The World Health Organization (WHO) estimates that 130–170 million people are chronically infected with the HCV, and is a leading cause of liver transplants.^[104]^ A number of detailed studies describing the design, structure–activity relationship studies, scale‐up synthesis, and clinical trials of novel macrocyclic HCV protease inhibitors have recently been published. Interestingly, despite a variety of macrocylization methods having been tried, one of the most frequently used—also in large‐scale production—was the RCM reaction (Figure 4). Ciluprevir, the first such macrocycle reported,^[105]^ failed in clinical trials; nevertheless, its synthesis remains important as the first commercially viable large‐scale RCM macrocyclization that influenced a number of subsequent synthetic approaches to other anti‐HCV macrocycles. The original scale‐up reaction leading to the formation of the Ciluprevir cyclic precursor was carried out at high dilution with 5–7 mol % of the catalyst **Ru8**. Key to a more economical process was the modification of the substrate structure by installation of a Boc protecting group on the proline NH amide fragment (marked in bold in Figure 4 a). This seemingly small change significantly reduced the ring strain, and allowed RCM at concentrations 10–20 times higher than those used previously. A switch to an EWG‐activated catalyst further improved the process economy, as only 0.1 mol % **Ru12** was needed to obtain a 93 % yield of **45**.^[106]^ In summary, the researchers at Boehringer Ingelheim put a lot of effort into optimizating the RCM process, not only focusing on practical issues but, importantly, understanding its reaction mechanism and kinetics.^[107]^ Thus, synthetic strategies optimized for Ciluprevir, such as controlling the effective molarity with N‐protecting groups, work not only in closely related cases, such as the formation of the macrocyclic core of BI201302,^[108]^ but were also useful in RCM leading to less related APIs, such as Simeprevir^[109]^ and others.

Researchers from GlaxoSmithKline and Anacor Pharmaceuticals Inc. reported the serendipitous discovery of a novel and potent HCV protease inhibitor as a by‐product in the synthesis of another antiviral molecule.^[110]^ Like some of the predecessors (e.g. Danoprevir), the macrocyclic urea derivative **46** was obtained in a high yield by the RCM reaction, this time in the presence of the Zhan‐1B catalyst under high‐dilution conditions (*c*=0.01 m, Figure 4 b) and with a high catalyst loading (20 mol %).^[110]^ The next example is the preparation of IDX316 (Figure 4 c).^[111]^ Instead of using the previously utilized strategy, in this case the metathesis reaction was implemented as the final step to generate the API. The evident advantage of late‐stage RCM is the shorter and less complicated synthetic route that has a lower cost contribution of the RCM step to the overall cost of the synthesis. The potential disadvantage is that removal of the toxic^[112]^ Ru catalyst at the API step can be more difficult and lead to contamination of the product with metal traces, especially as the RCM reaction was conducted with a relatively large loading of 1.25 mol % of the Zhan catalyst **Ru14**. After comprehensive studies on the removal of the catalyst on a kilogram scale, a combination of treatment with triphenylphosphine oxide and a dimercaptotriazine solid‐supported scavenger was utilized to reduce the Ru level to <10 ppm.^[111]^

Merck recently reported the multi‐kilogram synthesis of the MK6325 drug candidate, in which the RCM step was also made at the late stage of the synthesis, when the heteroaromatic part, typical for this class of molecules, had already been installed (Figure 4 d).^[113]^ Although MK6325 consists of two macrocyclic fragments, only one of them was formed by RCM. The metathesis‐based macrocyclization to afford **48** was effected in toluene at rather high dilution (0.06 m) by the slow addition of Zhan‐1B (0.45 mol %) at 80 °C, whereby the presence of benzoquinone was mandatory to inhibit isomerization of the C−C double bond.^[113]^

**Scheme 18:**
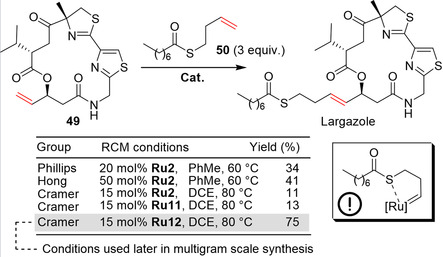
Discovery stage and larger scale synthesis of Largazole.

Not only has RCM been utilized in pharmaceutical R&D and scale‐up laboratories. Luesch and co‐workers disclosed at the beginning of 2008 the structure of Largazole, a novel peptide‐polyketite hybrid.^[114]^ It was isolated in trace amounts from a marine cyanobacteria of the genus *Symploca* (reclassified now as the new genus *Caldora penicillata*) collected at Key Largo, Florida. Largazole displays very potent growth inhibition activity in several transformed human and murine‐derived cell lines. Many research groups targeted the synthesis of Largazole, and these advances have been reviewed.^[115]^ Among them, the groups of Hong,^[116]^ Cramer,^[117]^ and Phillips^[118]^ ambitiously opted to introduce the complete thioester side chain by a cross‐metathesis reaction in the very last step of the synthesis (Scheme 18). Such an approach has a built‐in advantage of avoiding the need for several protection group manipulations, and an additional strategic benefit consists of the simplified generation of analogues (needed for SAR studies) just by exchange of the cross‐metathesis olefinic partner (e.g. **50**). However, as far as the original structure of Largazole is concerned, one must consider the risks related to cross‐metathesis with an olefin bearing a sulfur substituent in the chelating position (Scheme 18, insert). This constitutional difficulty was reflected in the high catalyst loading (20–50 mol %) used in all published syntheses and the modest yields obtained with second‐generation Grubbs and Hoveyda–Grubbs catalysts. The best solution found by Cramer and co‐workers was to use the more active nitro catalyst **Ru12**, which allowed Largazole to be obtained in an acceptable 75 % yield. Recently, Oceanyx Pharmaceuticals Inc. reported the development of the scaled‐up synthesis of Largazole by using Cramer's conditions for the crucial cross‐metathesis step and utilizing **Ru12** (Scheme 18).^[119]^ In the developed process, decagrams of Largazole were synthesized in an overall yield of 21 % for the longest linear sequence.

**Scheme 19:**
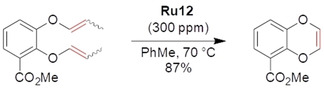
Synthesis of 1,4‐benzodioxane by RCM.

Researchers at Boehringer Ingelheim Pharmaceuticals and Astatech BioPharmaceutical Corporation, supported by experts on olefin metathesis from Apeiron Synthesis S.A., reported the stereoselective synthesis of substituted 1,4‐benzodioxanes that are structural motifs of several drugs.^[120]^ As the pharmaceutical industry has to keep the chemical production process as competitive and as cost‐effective as possible, a lot of effort was invested in optimizing each of the individual reaction steps. RCM of substrates containing vinyl ethers is known to be problematic, and a high loading of the second‐generation Grubbs catalyst (5–8 mol %) was previously reported for the synthesis of 1,4‐benzodioxines,^[121]^ which hindered the practicality of this transformation. Gratifyingly, amounts of nitro catalyst **Ru12** as low as 150 to 300 ppm were found to lead to the target 1,4‐benzodioxanes in >80 % yield (Scheme 19).

## 5 Production of Specialty Chemicals and Commodities, as well as Applications in Materials Science

**Scheme 20:**
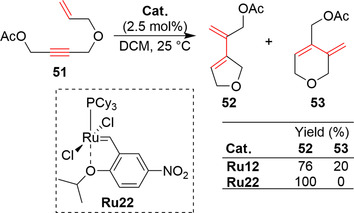
Utilization of enyne metathesis in the formation of five‐membered rings.

To the best of our knowledge, the nitro‐ and sulfonamide‐activated (Zhan‐1B) catalysts of the EWG‐activated family of catalysts are only commercially available as their first‐generation versions (L=PCy_3_). Although the Hoveyda–Grubbs catalysts with SIMes or SIPr ligands are undoubtedly more stable and active,[^18^, ^33^] there is a limited number of potential applications for the first generation of EWG‐activated catalysts. One of them is enyne metathesis of a certain class of substrates bearing an internal acetylenic bond.[^22^, ^122^] It was observed in this case that the use of second‐generation Grubbs or Hoveyda–Grubbs catalysts usually led to the formation of undesired products (e.g. **53**; Scheme 20). A similar lack of selectivity has been reported by Mori and co‐workers for the second‐generation Grubbs catalyst bearing an IMes ligand.^[123]^ Interestingly, the first‐generation nitro catalyst (**Ru22**) shows a high level of selectivity in this transformation, leading only to the formation of **52**. This observation is also true in the case of other enynes possessing an internal alkyne motif.[^22^, ^122^]

**Scheme 21:**
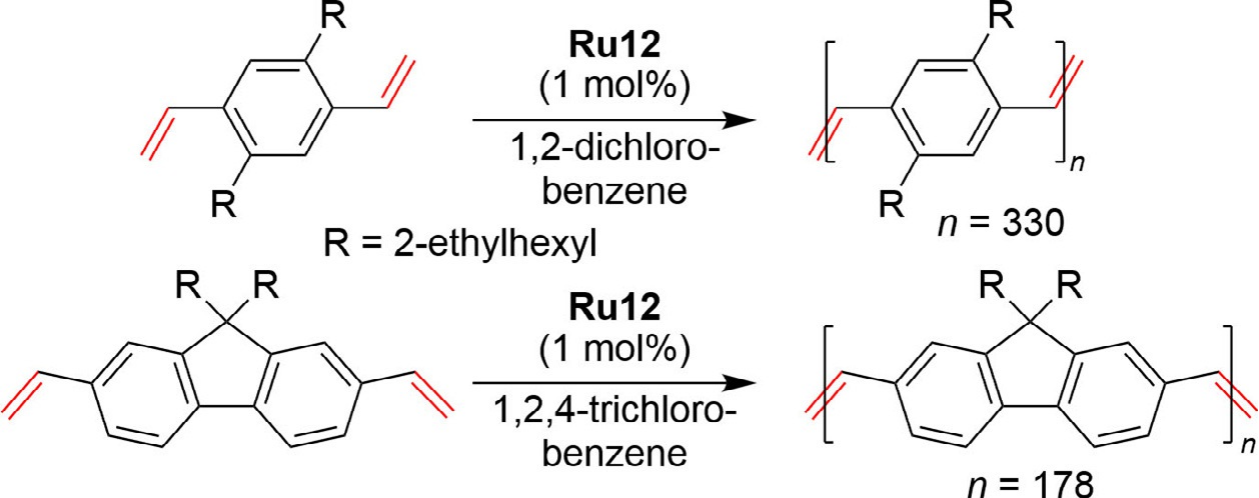
ADMET in the polymerization of divinylbenzene and divinylfluorene.

EWG‐activated catalysts have also been tried in ADMET polymerization (Scheme 1) for the synthesis of poly(*p*‐phenylenevinylenes) (PPVs)—important materials for applications in organic light‐emitting diodes (OLEDs) and organic photoconductors. In this study, selected divinylbenzene and divinylfluorene monomers were polymerized under vacuum using **Ru2** or **Ru12** olefin metathesis catalysts to afford PPVs as free‐standing films (Scheme 21).^[124]^ The nitro‐activated catalyst has also been used in the synthesis of selectively substituted indenes, which have potential applications in photovoltaics.^[125]^

**Scheme 22:**
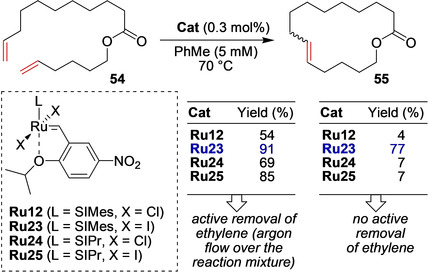
Influence of anionic ligands on RCM macrocylization.

An important increase in the scope of applications of the EWG‐activated catalysts was achieved through further modifications of the catalysts structure. The iodide‐containing nitro catalysts were synthesized by scientists from the company Apeiron Synthesis S.A. and applied in a number of challenging RCM and cross‐metathesis reactions, also in “green” solvents (Scheme 22).^[126]^ It was noted that the augmented steric hindrance in the vicinity of the Ru center (because of the higher ionic radius of the iodide ligands compared with chloride ligands) ensures the higher stability and robustness of the catalyst. This benefit was best illustrated under highly challenging conditions, such as in reactions with very low catalyst loading, in protic (MeOH, *i*PrOH) or in Lewis‐basic solvents (2‐MeTHF), or in the presence of various impurities or ethylene. The presence of ethylene (instantaneous removal of which is sometimes difficult to achieve under industrial large‐scale conditions) sometimes has a dramatic effect on the reaction yield and selectivity, as illustrated in Scheme 22. The increased stability of the ruthenium methylidenes generated from **Ru23** makes this catalyst especially suitable for the macrocyclization of unbiased dienes, such as **54** with low effective molarity, also in the presence of ethylene.^[126]^

**Figure 5:**
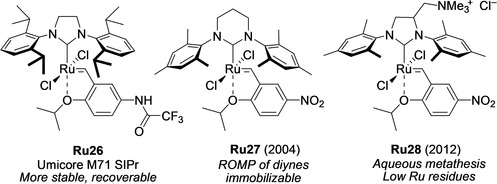
EWG‐activated catalysts with modified NHC ligands.

Another potentially important direction in the further improvement of EWG‐activated catalysts lies in modification of the NHC ligand. Although both SIMes (initiating faster) and SIPr (more stable)[^33^, ^127^] versions of the leading EWG catalysts (M71, M73, and nitro) are commercially available, other NHC modifications are less obvious, but some of them are very interesting and will be described here. Buchmeiser and co‐workers reported the synthesis of nitro catalyst **Ru27** bearing a 1,3‐bis(2,4,6‐trimethylphenyl)‐3,4,5,6‐tetrahydropyrimidin‐2‐ylidene ligand (Figure 5). This catalyst was tested in a number of RCM, cross‐metathesis, and ROMP transformations, and was also immobilized on a solid support, which led to a very low Ru contamination of the products.^[128]^ Apeiron Synthesis S.A. developed a unique class of self‐scavenging olefin metathesis catalysts bearing a polar quaternary ammonium group installed on the backbone of standard SIMes or SIPr ligands.^[16]^ The presence of this group allows efficient separation of ruthenium impurities after the reaction. A representative member of this class is complex **Ru28** ^[129]^ (Figure 5), which in addition is water soluble and can also promote olefin metathesis in aqueous media.[^14^, ^130^] The application of catalyst **Ru28** led to products that exhibited low ruthenium contamination levels after a simple and inexpensive purification step, consisting only of water extraction or filtration through a short pad of silica.^[129]^

**Scheme 23:**
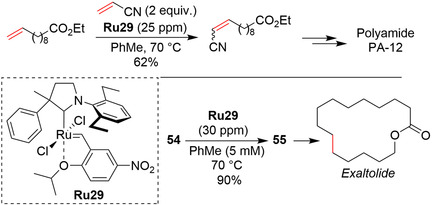
Applications of UltraNitroCat **Ru29** at low loading.

The active Ru species generated from ruthenium complexes bearing standard SIMes or even SIPr NHC ligands exhibit limited stability under certain demanding conditions (high dilution, high temperature, presence of ethylene).^[103]^ As a consequence, industrial implementation of modern expensive metathesis catalysts in a large‐scale production of commodity materials (where the low product price excludes technically complicated and cost‐intensive approaches) is problematic. RCM production of macrocyclic musks, selective homometathesis of α‐olefins, or ethenolysis of bio‐sourced fatty oils are examples of processes were the great potential of metathesis is hampered by the low stability of the available catalysts. Over the past 19 years, a large number of ruthenium complexes bearing NHC ligands have been obtained, but most of them cannot be used at ppm levels in the above‐mentioned processes because of their insufficient lifetime under demanding reaction conditions in the presence of various impurities. Based on excellent results reported by Bertrand, Grubbs, and co‐workers on cyclic alkyl amino carbene (CAAC) ligands,^[131]^ Skowerski and co‐workers disclosed the EWG‐activated ruthenium CAAC complex **Ru29**, which promoted the difficult RCM macrocyclization leading to a musk‐smelling lactone at a catalyst loading of 30 ppm, and cross‐metathesis of acrylonitrile at 25 ppm (Scheme 23).^[132]^ The latter result, in particular, which was obtained in cooperation with the company Arkema, is of interest, as acrylonitrile was for years considered to be a very difficult cross‐metathesis partner and reactions with this partner usually required the use of industrially unacceptably high amounts of metal.[^133^, ^134^, ^135^]

In an in‐depth study, Nascimento and Fogg explained the fundamental reasons for the remarkable productivity of **Ru29**, stressing the fact that while the CAAC catalysts are more resistant to α‐elimination, they are very susceptible to bimolecular decomposition—a well‐known path of destruction for many Ru catalysts. Importantly, however, because the CAAC catalysts can be used at very low catalyst loading, the bimolecular decomposition is inhibited under these conditions, thereby making them extraordinarily productive.^[136]^

The translation of a reaction from the laboratory to process scale using traditional batch techniques is sometimes very challenging. In the specific case of olefin metathesis, a strong dependence of the reaction yield on the reactor design and scale was noted many times.^[103]^ Exploring this area, researchers from Snapdragon Chemistry Inc. and Massachusetts Institute of Technology developed a special continuous‐flow reactor featuring a membrane sheet‐in‐frame pervaporation module that enables effective removal of ethylene. Under these conditions, the diiodo complex **Ru23** and UltraNitroCat **Ru29** gave particularly good results in RCM macrocyclizations relevant to the fragrance industry.^[137]^

**Scheme 24:**
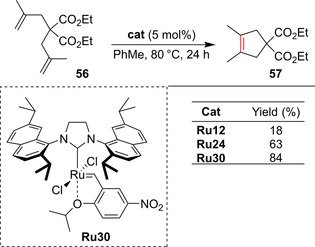
Comparison of nitro catalysts bearing different NHC ligands in the formation of tetrasubstituted olefins.

Recently, Dorta and co‐workers reported the preparation of nitro catalysts bearing sterically augmented NHC ligands.^[138]^ Importantly, complex **Ru30** demonstrated quite good activity in the formation of tetrasubstituted C−C double bonds, the reaction which was traditionally the Achilles heel of nitro catalyst **Ru12** (Scheme 24).[^21^, ^22^]

We hope that the collected examples illustrate the potential of the application of EWG‐activated Hoveyda–Grubbs catalysts in the synthesis of various building blocks and fine‐chemicals, as well as in materials science.

## 6 Practical Considerations, Outlook, and Perspectives

The specialized EWG‐activated catalysts, together with the already very successful classical Schrock and Grubbs‐type catalysts, constitute a powerful toolkit that allows synthetic chemists to perform even very challenging metathesis transformations. However, the variety of metathesis catalysts now commercially available makes the proper choice of the catalyst a true problem. The data reported herein demonstrate that great care must be taken when choosing the appropriate catalyst for a given metathesis reaction. The avid reader certainly has identified a number of examples where more than one metathesis catalyst type has been used in a given total synthesis (e.g. one catalyst for cross‐metathesis and the other one later for RCM; see Section 3.1).[^57^, ^63^, ^85^, ^86^] Interestingly, and in contrast to a growing number of applications of EWG‐activated catalysts in total synthesis and medicinal chemistry, these complexes have found only limited use in polymer production through ROMP, where other catalyst types dominate.[^139^, ^140^, ^141^, ^142^, ^143^] We can only repeat what we stated already in 2008: different metathesis catalysts prove to be optimal for different applications and no single catalyst can outperform all others in all cases.^[72]^ Therefore, during optimization of especially important (or industrial) metathesis processes, it is suggested to screen all major types of catalysts available, on one's own or with the help of metathesis experts. Our long‐term experience advises that more close cooperation between synthetic chemists (“end‐users”) and the catalyst developers or manufactures can substantially speed‐up and smooth the costly optimization phase, especially in the case of complicated industrial projects. For example, the typical loading of a metathesis catalyst used in academia‐published total syntheses of natural products is from 5 to 25 mol % or even higher (up to 100 mol %).^[144]^ This is, of course, fully understandable in academic research, and even justified at the early stages of the industrial R&D work, but sooner or later the loading must be substantially reduced to make the production process economically viable. Some examples where the amount of catalyst used was reduced from a multiple molar percent to its decimal fractions or even to ppm levels was achieved, thanks to the cooperation with the catalyst producer or metathesis expert, have been presented in this Review.[^98^, ^106^, ^120^] In this context, recent results by Nascimento and Fogg are quoted again, as they were able to prove that lowering the loading of the CAAC nitro catalyst is not only favorable from an economic point of view, but in fact it also increases the catalyst's stability, by inhibiting the bimolecular decomposition pathway.^[136]^ This result shows yet again the importance of fundamental studies to deepen our understanding of the factors that influence the stability and activity of catalysts. In the practice of total synthesis leading to complex polyfunctional natural products and in medicinal chemistry, the prediction of the most optimal catalyst for a given substrate is not an otiose question but rather a serious problem. We hope that a better understanding of the initiation and decomposition mechanisms of catalysts will help to solve this riddle.

The importance of the proper choice of a solvent (both classical petroleum‐based solvents and more eco‐friendly “green” ones are available nowadays),^[145]^ the beneficial influence of various additives (e.g. phenols and quinones),^[50]^ the surprising effect of fluorinated aromatic solvents,[^46^, ^47^, ^89^, ^90^] and many other “enabling techniques”^[146]^ have also been identified. Importantly, it is not only through the use of such sophisticated techniques, but even changing the simplest reaction parameters, such as temperature and concentration, switching to a batch‐wise addition of the catalyst, or just more efficient removal of ethylene, can sometimes significantly improve the outcome of the metathesis reaction.

In a summary, we have tried to convince the reader that EWG‐activated Ru catalysts have enabled, and will continue to enable, syntheses of various chemical molecules in many fields of organic and medicinal chemistry.

## Conflict of interest

The authors declare no conflict of interest, but K.G. is an advisory board member of the Apeiron Synthesis company, the producer of some catalysts described herewith.

## Biographical Information

*Anna Kajetanowicz received her Master degree from Warsaw University of Technology and PhD from the Institute of Organic Chemistry PAS. She held two postdoctoral fellowships, first in Prof. Karol Grela's group in Warsaw and the second with Prof. Thomas R. Ward in Basel. She then returned to IChO PAS and in 2015 she moved to the University of Warsaw. Since 2018 she has been deputy director of the Laboratory of Organometallic Synthesis*.

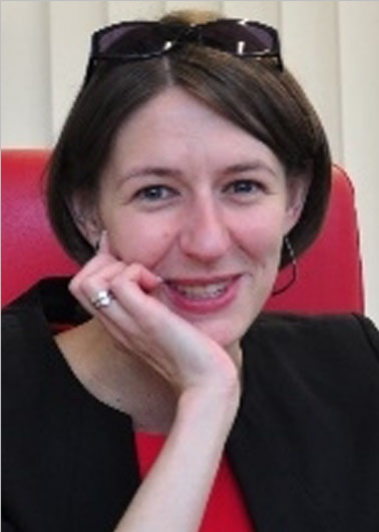

## Biographical Information

*Karol Grela received his Master degree from Warsaw University of Technology, and PhD from the Institute of Organic Chemistry PAS. As an Alexander von Humboldt Scholar, he spent one year at the Max‐Planck‐Institut für Kohlenforschung in the laboratories of Prof. Alois Fürstner. He then returned to Warsaw, and completed his Habilitation in 2003. In 2008 he was promoted to Full Professor. Since 2008, he has been a Director of a newly formed group at the Biological and Chemical Research Centre of the Faculty of Chemistry, University of Warsaw*.

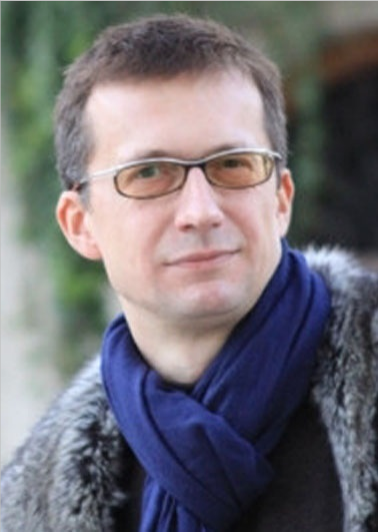

## Supporting information

As a service to our authors and readers, this journal provides supporting information supplied by the authors. Such materials are peer reviewed and may be re‐organized for online delivery, but are not copy‐edited or typeset. Technical support issues arising from supporting information (other than missing files) should be addressed to the authors.

Supplementary
