# Supplementary material for: Nitro and Other Electron Withdrawing Group Activated Ruthenium Catalysts for Olefin Metathesis Reactions
Source: Angew Chem Int Ed Engl. 2020 Dec 3;60(25):13738–56. doi: 10.1002/anie.202008150 (PMC8246989; doi:10.1002/anie.202008150)
Supplement: Supplementary file 1 — Supplementary [file ANIE-60-13738-s001.pdf]

Author Contributions

A.K. Writing - Original Draft: Supporting; Writing - Review & Editing: Equal.
